# Assessment of concentration and distribution of total mercury and polychlorinated biphenyls in Green Bay, Wisconsin, USA

**DOI:** 10.1007/s11356-021-16417-6

**Published:** 2021-09-29

**Authors:** Marcia R. Silva, Alice Lecus, Chad Haehle, David Garman, Shelby Brunner

**Affiliations:** 1 Water Technology Accelerator (WaTA), University of Wisconsin-Milwaukee, 247 W. Freshwater Way, Milwaukee, Wisconsin 53204 USA; 2 Centre for Infrastructure Engineering, Western Sydney University, 56 Second Avenue, Kingswood, NSW 2747 Australia; 3 UCAR-The University Corporation of Atmospheric Research, Silver Spring, MD 20910 USA

**Keywords:** Total mercury, Sediments, Green Bay, PCBs, Aroclor 1242, Pore water

## Abstract

Under the 1987 Great Lakes Water Quality Agreement, the lower Green Bay and Fox River estuary have been labeled as areas of concern due to the contamination of mercury and polychlorinated biphenyls (PCBs) from industrialization. These pollutants pose substantial health and environmental hazards for the Green Bay region. The PCBs reported in this region, including Aroclor 1242, are known to trigger carcinogenic responses in animals and mercury targets the central nervous system and vital organs. Furthermore, these compounds are extremely difficult to remove from the environment once introduced. Extensive remedial actions have been implemented including dredging sediments in the Lower Fox River from DePere to Green Bay. The purpose of this study is to assess the concentration and distribution of Aroclor 1242 and total mercury in the Green Bay region sediments and pore waters and to assess the impact of interventions and the natural rates of change previously found.

**Supplementary Information:**

The online version contains supplementary material available at 10.1007/s11356-021-16417-6.

## Introduction

The Green Bay watershed, including the Fox-Wolf River in northeastern Wisconsin, is approximately 16,000 km2 and is dominated by the Fox River accounting for one-third of the entire Lake Michigan (LM) drainage basin (EPA 1989; Yin et al. 2016). The land has been utilized for urban, agricultural, and industrial purposes since the mid-1800’s when the first paper mill was built in 1865 (Yin et al. 2016). In 1995 the Lower Fox River sub-basin located between Lake Winnebago and Green Bay had the highest density of paper mills in the world (Hurley et al. 1998). This industrial activity involved the use of polychlorinated biphenyls (PCBs) and total mercury (HgT), which were then discharged into local water ways and substantially impairing both water and sediment. It was reported that the locations with the highest concentrations of mercury were associated with the discharge of municipal wastewater into the environment (Glass et al. 1990a). This is also the case for PCBs, municipal waste was also monitored source of contamination (EPA 1989).

In response to PCB, mercury, and additional contaminant issues, the Great Lakes Water Quality Agreement (GLWQA), in 1987, directed the International Joint Commission (IJC) to identify areas of concern (AOC) where beneficial water sources were impaired. IJC surveyed 42 locations, including the Lower Fox River and Green Bay. This AOC extends from the DePere Dam, seven miles from the mouth of the Fox River, to the Long-tail Peninsula roughly three miles 5 km into Green Bay. The U.S. Environmental Protection Agency (EPA) directed the states where an AOC was identified to develop a remedial action plan (RAP) for the purpose of improving the water and aquatic habitat quality (Gurtner-Zimmermann 1996). The Green Bay RAP was among the first to be submitted to the IJC in 1988. At the time, this was one of two plans to meet the ecosystem-oriented criteria.

Total mercury exists primarily as the inorganic mercuric ion, Hg(II), in sediment and water; elemental mercury, Hg(0), in the atmosphere; and organic methylmercury (Agency 1977). Considered a global contaminant, mercury, a naturally occurring element, can undergo long-range atmospheric transport in the form of Hg(0). Hg(II), dissolved or adsorbed onto particles in droplets, can also travel by means of atmospheric waters and is readily deposited on the earth’s surface. In water and sediment, total mercury mainly consists of these Hg(II) compounds, both inorganic and organic. Hg(0) is the second most common form found in water and sediment and plays a role in the exchange of mercury between the atmosphere, water and terrestrial surfaces. Finally, the formation of methylmercury, typically less than 10% of HgT, results in bioaccumulation and high concentrations in the food web.

The high levels of HgT found near Green Bay is of greatest concern due to its adverse health effects on humans and wildlife. Acute mercury exposure, from organic or inorganic mercury, can lead to permanent nervous system damage, immune malfunction, myocardial infarction, and damage of the brain (Rice et al. 2014). In addition, MeHg bioaccumulates in organisms persisting as a neurotoxin. While high exposure to humans is rare today, fish contamination remains a concern due to the potential biomagnification. Generally, the severity of mercury poisoning depends on the magnitude of exposure and is the reason why fish tissue is evaluated for these toxins. Tissue samples are examined to determine the levels of contamination and whether a public fish advisory is required. While mercury is a naturally occurring element, the high levels of contamination found near Green Bay are a result of both natural and anthropogenic sources (Achman et al. 1993). Anthropogenic sources include point sources, such as combustion and manufacturing industries, in addition to landfills, mine waste, and sewage sludge (Agency 1997). Industrialization, specifically paper mills as mentioned, has substantially impaired the health of the Fox River and the Green Bay region leaving high levels of contamination as a result. To this day, despite intensive remedial efforts, water recreational use is extremely limited, fish consumption warnings remain in place, and the Lower Fox River tributary has yet to be delisted as an AOC due to the persistent nature of mercury. A Green Bay and Fox River mass balance study determined the extent of HgT contamination in the region (Gurtner-Zimmermann 1996) for 11 tributaries in Lake Michigan. It was determined that the Fox River estuary possessed the highest mean HgT concentrations in water with 27.9 ng·L-1 compared to 1.05 to 10.3 ng·L-1 found in the other tributaries (Hurley et al. 1998). Sediment tested near the DePere Dam had a HgT concentration as high as 7.400 mg·kg-1 (Hurley et al. 1998).

In addition to mercury contamination, the Green Bay lake, in addition to the Fox River, has a legacy for PCBs with levels as high as 710 mg·kg-1 in sediment (USGS 2000). In the late 1980’s the U.S. EPA published their plans to begin a mass balance study that focused on PCB contamination in the Fox River and the Green Bay lake (EPA 1989). The purpose of the study was to establish a baseline for PCB concentrations in water and sediment. The high levels of PCBs have affected the environment and wildlife so much that local officials had to issue advisories for game fishing, closing commercial fishing for certain species all together (EPA 1989). Fish consuming birds in the region were also reported to be developing deformities in addition to other reproductive problems associated with PCBs. PCBs can accumulate in aquatic species’ tissues in high concentrations. In addition to their persistence, they are also very toxic compounds that show carcinogenic responses in both humans and animals (ATSDR 2011). The PCB emphasized in this study, Aroclor 1242, was chosen because it was most commonly used in the affected region (Cacela et al. 2002). The general chemical structure for a PCB is composed of two benzene rings with varying numbers (between 1 and 10) of chlorine atoms attached. Aroclor 1242 has a chlorine composition of 42% which can be derived from the last two digits of its name. These compounds are very stable as a result of aromaticity and can take up to decades to naturally decompose. In aqueous solution, the solubility limits for range from 1.00 x 10-5 ng·L-1 to 3.40 x 10-5 ng·L-1 at 24°C and 25°C respectively (ATSDR 2017). Because of low solubility, the affinity to solid materials, including sediment and biological tissue, is much higher.

In order to resolve this problem, officials in Green Bay initiated a cleanup effort using a process known as dredging. Dredging is a process where large quantities of contaminated sediment is physically removed from the floor of a lake or river in order to remove the contaminants. While dredging was intended to reduce the concentrations of these toxic materials from the Fox River, it was found that it can lead to the resuspension and transportation of contaminated sediment (USGS 2000). This method is also incredibly expensive, approximately US$ 100 million has already been spent dredging the Fox River to the DePere Dam with an anticipated US$ 1.2 billion for future clean-up (Bergquist 2019). Despite the many issues associated with this method, prior studies have shown that the levels of HgT and PCBs should have been reduced. This research aims to 1) investigate the concentration levels of PCBs (Aroclor 1242) and HgT in Green Bay sediments 2) determine the partitioning coefficient to assess the risk for transport of these pollutants, and 3) compare the residual concentrations with those expected from natural decay and removal processes. It is our hypothesis that the current concentrations for HgT and PCBs will be similar o those found historically due to their persistence in the environment.

## Materials and methods

### Study site description

The Green Bay watershed, including the Fox-Wolf River in northeastern Wisconsin, is approximately 16,000 km2 and is dominated by the Fox River accounting for one-third of the entire Lake Michigan (LM) drainage basin (EPA 1989; Yin et al. 2016). The hydrological patterns found in the embayment divide the lake into two distinct regions, the inner and outer bay (Cacela et al. 2002). The most substantial input, the Fox River, located at the south western side of the inner bay contributes to a significant amount of sediment deposition along the Eastern regions of the bay.

### Sediment sampling

Sediment samples were collected in Summer 2016 using a Soutar-type box corer (1000 cm^2^). After inspection to determine that it was an undisturbed sample, overlying water > 1 cm was removed by slow side drainage. The remainder surficial water was removed by suction hose. The sediment was removed from the corer at discrete intervals by vacuum using a custom-built device (Klump 2015). Sediment from a 1 or 2 cm layer was removed to Buchner flasks before transferring to weighed, clean glass jars and was stored at 4°C before analysis.

### Mercury analysis

Sediment samples were centrifuged 4000 rpm for 5 min to obtain the pore water. Water was filtered sterilized (0.2 μm Millipore filter), oxidized with BrCl. In this procedure, the samples are oxidized with 1% (v/v) BrCl, if it is a clear liquid, or 2% (v/v) BrCl if there is particulate or discoloration in the liquid. Sediment was digested (4 h in 10 mL aqua regia, 4:1 HCl: HNO_3_). The digestate was filtered and oxidized as the pore water. All reagents and chemicals used were reagent grade unless otherwise indicated.

Sediment and pore water samples for HgT were measured using cold vapor atomic fluorescence spectroscopy (CVAFS) in accordance with US EPA Method 1631 (EPA 2002). Analytical CRM standards at a concentration of 1ppm HgT in 2% (v/v) nitric acid were purchased from Brooks Rand. The instrument Brooks Rand MERX-T detection limits for this method are 0.2 to 400 ng·L^-1^ for a 25-mL sample. Laboratory blanks of E-Pure water were used for quality control measures. In addition, two blanks were analyzed after every 10 samples to confirm that analytical techniques did not introduce measurable contamination.

### Pore PCB analysis

Sediment samples were centrifuged 4000 rpm for 5 min to separate the aqueous component from the solid. All pore water samples were extracted using the EPA 608 method (EPA 1984). Extractions were performed using a separatory funnel and were concentrated using a Kuderna-Danish (KD) concentrating apparatus. Sediment samples were extracted following the microwave extraction procedure outlined in EPA 3546 (EPA 2007). This method was used in place of the Soxhlet extraction method to reduce the waste of solvents and to reduce extraction time. All extractions were performed in a pressure sealed extraction vials using a CEM Mars 6 microwave. Three Teflon solvent extraction vessels were purchased from CEM. The sample extracts were then KD concentrated down to a final volume of less than 10mL. All reagents and chemicals used were reagent grade unless otherwise indicated.

All PCB samples were analyzed using a Shimadzu GCMS QP-2010 Ultra with DI probe. Aroclor 1242 (neat) was purchased from Ultra Scientific Analytical Solutions as a reference material (RM). The instrument was set to analyze the samples at 250°C for 21 min. Standards were prepared by dissolving pure Aroclor 1242 in reagent grade hexane. Hexane blanks were analyzed every 10 samples to check for solvent contamination and for carryover contamination between samples. A column bake method was run at the beginning of each analysis to reduce contamination. Percent recoveries for sediment samples were performed by using rinsed and extracted sediment which was dried and spiked with a known concentration of Aroclor standard. Pore water sample recovery was performed by extracting an aqueous solution prepared at the maximum solubility and an extraction of a 1:1 dilution of the solution. Peak areas for PCBs were obtained by taking the sum of all the peaks of interest after the retention time of 10 min.

### Distribution coefficient

Solid/liquid distribution coefficients (*K*_d_) are used to indicate the mobility of a contaminate in an environment. *K*_d_ (L kg^-1^) was calculated to determine distribution coefficients for HgT and Aroclor 1242 samples as follows in Equation 1:

$$ {K}_d=\frac{S}{D} $$

where *S* = concentration in the solid phase (mg kg^-1^) and *D* = concentration in the liquid phase (mg L^-1^).

### Geo-statistic interpolation and statistical analysis

Sample maps were generated using ArcGIS Pro 2.4.0 (Environmental System Research Institute (ESRI), Redlands, Canada. Samples were plotted by sample site and concentration, using a segmented 3-color scheme to indicate concentration range for PCB and mercury samples. The ESRI light grey canvas basemap was used to generate all maps. Principal component analysis (PCA) was carried out to provide a greater understanding of sample trends. All calculations for PCA were performed using SigmaPlot, version 14.0.

## Results and discussion

**Fig. 1:**
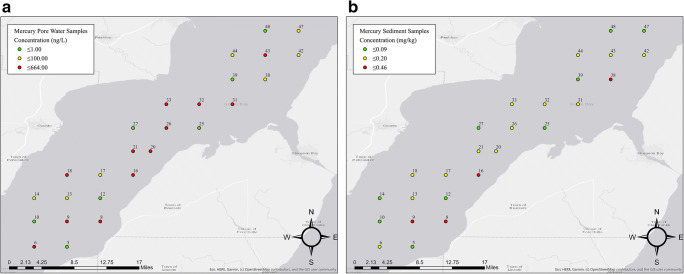
**a** Lower Green Bay HgT concentrations (ng·L^-1^) for pore water (**b**) lower Green Bay HgT concentrations (mg·kg^-1^) for solid matrix

HgT pore water concentrations range from 6.39 (ID 14) to 664 ng·L^-1^ (ID 32) as shown in (Fig. 1) and in (Table 1) with a mean of 175 ng·L^-1^. The mean sediment HgT concentration of 0.17 mg·kg^-1^ obtained from this study is lower when compared with the historical sediment concentrations taken from the Fox River, where the lowest mean sample was 0.97 mg·kg^-1^ in 1994 (Yin et al. 2016). Additionally, the mean HgT pore water concentrations from the Green Bay sites sampled in this study was a high of 175 ng·L^-1^, where the highest mean HgT pore water concentration from the Fox River was only 27.9 ng·L^-1^ in 1994 (Hurley et al. 1998). The sediment samples from the Lower Fox River have a much higher concentration of HgT than samples collected further out into the bay. A maximum concentration of 4.20 mg·kg^-1^ HgT compared to 0.460 mg·kg^-1^ HgT for the lower Green Bay. However, the opposite occurs when comparing the pore water samples, where the maximum concentration for Green Bay is 664 ng·L^-1^ compared to 182 ng·L^-1^ for the Fox River.

**Table 1.** Lower Green Bay HgT and PCB concentrations for solid matrix, pore water, and distribution coefficients. The results show much higher concentrations in sediment for PCBs than for mercury and that collection site 9 corresponds to the location with the highest concentration for both.

GB sample	PCBs			Mercury		
	Sediment conc. (mg**·**kg^-1^)	Pore water conc. (ng·L^-1^)	Distribution coefficient	Sediment conc. (mg**·**kg^-1^)	Pore water conc. (ng·L^-1^)	Distribution coefficient
5	7.83	0.00	0.00	-	-	-
6	2.91	0.299	9.76×10^6^	0.164	128	1.28×10^3^
7	7.35	1.270	5.78×10^6^	-	-	-
8	162.18	0.263	6.17×10^8^	0.244	124	1.97×10^3^
9	295.28	0.360	8.21×10^8^	0.456	414	1.10×10^3^
10	6.45	0.666	9.68×10^6^	-	-	-
11	21.05	0.827	2.55×10^7^	-	-	-
12	6.92	0.336	2.06×10^7^	-	-	-
13	3.83	0.223	1.72×10^7^	0.131	11.9	1.10×10^4^
14	7.39	0.235	3.14×10^7^	0.02033	6.39	3.18×10^3^
16	1.63	0.250	6.53×10^6^	0.428	315	1.36×10^3^
17	5.82	0.344	1.69×10^7^	0.09160	9.48	9.67×10^3^
18	3.66	0.542	6.75×10^6^	0.148	127	1.17×10^3^
20	0.87	0.991	8.76×10^5^	0.168	115	1.46×10^3^
21	1.38	0.227	6.10×10^6^	0.186	126	1.47×10^3^
25	5.11	0.182	2.81×10^7^	-	-	-
26	1.47	0.280	5.26×10^6^	0.108	441	2.45×10^2^
27	2.00	0.333	6.01×10^6^	0.0403	-	-
31	0.26	0.681	3.76E×10^5^	0.106	167	5.64×10^2^
32	1.40	0.299	4.68×10^6^	0.109	664	1.64×10^2^
33	0.84	0.282	2.99×10^6^	0.120	148	8.09×10^2^
38	0.62	0.000	0.00	0.460	55.7	8.26×10^3^
39	1.90	0.171	1.11×10^7^	-	-	-
42	0.72	0.290	2.48×10^6^	0.117	79.8	1.62×10^3^
43	1.47	0.277	5.31×10^6^	0.115	346	3.33×10^2^
44	3.88	0.192	2.02×10^7^	0.112	30.5	3.66×10^3^
47	5.68	0.872	6.51×10^6^	0.0662	18.5	3.58×10^3^
48	1.33	0.257	5.17×10^6^	-	-	-

**Fig. 2:**
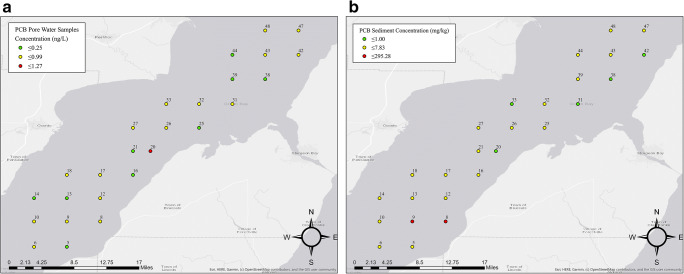
**a** Concentration of Aroclor 1242 in the lower Green Bay (ng·L^-1^) for pore water samples. **b** Concentration of Aroclor 1242 in the lower Green Bay (mg**·**kg^-1^) for sediment samples

Data obtained from PCB samples in both pore water and sediment show concentrations ranging from 0.99 to 295.85 mg·kg^-1^ for the lower Green Bay (Fig. 2) and (Table 1). These results are comparable to historical data from samples collected in 2002 where concentrations ranged from non-detectable to 30 mg·kg^-1^ (WDNR 2012). The results from collection sites 8 and 9 show similar sample concentrations reported in one study in 1999 (USGS 2000) where the maximum concentration was 710 mg·kg^-1^. Examining the map of the PCB distribution in the lower Green Bay region shows the highest concentrations along the southern region of the collection site. These results are consistent with the proximity to the known source—the Fox River. The peak response was very low in the pore water samples and only four peaks could be used for the peak integration for PCBs. The low peak response was attributed to the low solubility of Aroclor 1242. The way that the pore water samples were quantified has a high degree of error associated because the concentrations were either below the limit of detection or very close to it. The contribution of these samples to the total amount of Aroclor 1242 is negligible in comparison with the sediment samples, as they have substantially higher concentrations. Sediment samples were processed by integrating a maximum of 100 peaks within the noise range criteria. Many of the samples showed a large peak around 15 min that was not related to the compound of interest and was therefore excluded from the peak integration. The distribution coefficients for Hg and PCBs were calculated in Table 1, showing greater mobility for Hg than for PCBs. Pore water diffusion rates of 0.4cm/day were reported for PCBs from a study conducted in 1994 also indicate limited mobility (Velleux and Endicott 1994). It should be noted that despite higher mobility than PCBs, most Hg samples showed low mobility.

Of the sites sampled in this study, 16 were found to have a concentration greater than 12 ng·L^-1^ HgT in pore water and two sites were found to have more than 162 mg·kg^-1^ of PCB in sediment. Most of these sites are toward the eastern side of Green Bay. Site 9, which is located near the southeast edge of the sampling area and subject to the greatest amount of Fox River inputs, contains the highest concentration of both Aroclor 1242 and the second highest concentration of mercury. Relative to the time of sample collection, the most recent phase of remediation began in 2009 and involved dredging, dredging and capping, and sand covering. This phase includes the area from the mouth of the Fox River to 13 miles upstream where further research may prove remedial success (WDNR 2012). When comparing the Green Bay concentrations with historical data (Table 2) from the Fox River, the most notable difference in mercury concentrations is the distribution coefficients. Typically, past data shows consistently higher concentrations of HgT in sediment located throughout the Lower Fox River than in Green Bay, while the opposite is found to be true for mercury concentrations in pore water.

**Table 2.** Historical concentrations of mercury in Fox River and Green Bay.

Location	Sediment (mg**·**kg^-1^)		Pore water (ng·L^-1^)			
	Range	Mean	Range	Mean	Sampling year	Source
Influent from Fox river	-	-	20–40	-	1988	(Glass et al. 1990b)
Lower Green Bay (0 to 30 km from mouth of Fox River)	-	-	< 12	-	1988	(Glass et al. 1990b)
DePere Dam	-	-	60	-	1990	(Glass et al. 1990b)
DePere Dam	up to 7.4	-	-	-	1993	(Hurley et al. 1998)
Downstream of DePere Dam	0.07–2.60	1.04	-	-	1993	(Hurley et al. 1998)
Kimberly	0.23–0.76	0.44	-	-	1993	(Hurley et al. 1998)
Wrightsown	0.34–0.88	0.61	-	-	1993	(Hurley et al. 1998)
Fox River	-	0.97	-	-	1994	(Yin et al. 2016)
Mouth of Fox River	up to 4.190	-	-	-	1994	(Yin et al. 2016)
Mouth of Fox River	-	-	7.81 to 182	27.9	1994	(Hurley et al. 1998)
DePere Dam	0.68–1.31	0.97	-	-	1995	(Hurley et al. 1998)
Lake Winnebago	-	-	<2	-	1995	(Hurley et al. 1998)
Mouth of Fox River	-	-	-	Median of 19.3	1995	(Hurley et al. 1998)
Wrightsown	-		4.2, 7.1	-	1995	(Hurley et al. 1998)
Appleton to Little Rapids Reach	-	2.143	-	-	1999	(ThermoRetec 1999)
DePere to Green Bay Reach	-	1.151	-	-	1999	(ThermoRetec 1999)
Little Lake Butte des Morts Reach	-	1.177	-	-	1999	(ThermoRetec 1999)
Little Rapids to DePere Reach	-	2.339	-	-	1999	(ThermoRetec 1999)
Lake Michigan - Nearshore	-	-	0.59 ± 0.12	-	2006	(Dove et al. 2012)
Lake Michigan - Open			0.49 ± 0.09		2006	(Dove et al. 2012)
Lower Green Bay	0.020–0.466	0.17	5.99-671	175	2016	This Study

When compared to the results obtained in this study, historical data for PCB concentrations in the Fox River shows an unexpected trend (Table 3). The data suggests that the concentration has substantially increased from 1976 to 2001 instead of decreasing over time. The highest concentration found in the 2001 study was 710 mg·kg^-1^, which is comparable to the highest concentration 295.28 mg·kg^-1^ found along the east side of the Green Bay lake in this study. While the PCB degradation rates are extremely variable, the natural rate of decomposition appears to be slowing down (Stratus 2000). The decline in PCB concentration in Green Bay was the most significant in the 1970s and 1980s but started slowing down shortly after. The rate of PCB degradation was projected to continue this pattern in the future. One group evaluated the Fox River during a four-month dredging operation that revealed consistent increase in concentration of total PCBs downstream (USGS 2000). This was explained to be due to increasing concentration of dissolved PCB from resuspending the contaminated sediment. It is important to note that besides dredging, there are other potential causes of resuspension. The DePere dam’s release of water (EPA 2017) causes sediment to resuspend. In addition, there are seasonal impacts (peak flows) that will also cause resuspension (Hurley et al. 1998).

**Table 3.** Historical concentrations of PCBs in Fox River sediments since 1976, based on findings in other studies. This information provides a comparison between previous PCB levels to what is observed today in this study. Concentrations reported for 2001 are higher than what has been previously reported.

Location	Sampling year	Low concentration (mg·kg^-1^)	High concentration (mg·kg^-1^)	References
Lake Winnebago	2001	Non-detectable	0.036	(ATSDR 2011)
Little Lake Butte des Morts	2001	2.0×10^-3^	222.722	(ATSDR 2011)
Appleton to Little Rapids	2001	Non-detectable	185.560	(ATSDR 2011)
Little Rapids to DePere Dam	2001	3.0×10^-3^	54.000	(ATSDR 2011)
De PereDam to mouth of River	2001	4.0×10^-3^	710.000	(ATSDR 2011)
Little Lake Butte des Morts	1976	Not applicable	0.027	(USFWS 2016)
Appleton	1987–1988	Not applicable	1.37×10^-4^	(USFWS 2016)
Downstream of DePere Dam	1976	Not applicable	7.5×10^-3^	(USFWS 2016)
DePere	1989–1990	Not applicable	0.115	(USFWS 2016)
Mouth of Fox River	1976	Not applicable	0.0108	(USFWS 2016)
Mouth of Fox River	1980–1983	Not applicable	2.62×10^-4^	(USFWS 2016)
Mouth of Fox River	1989–1990	Not applicable	1.52×10^-4^	(USFWS 2016)

The primary source for Hg and PCB pollution into the Green Bay Lake is from the Fox River. The dominating mechanism associated with Hg transport is attributed to the resuspension of bottom sediments in the Fox River, where HgT concentrations increase closer to the DePerer Dam (Hurley et al. 1998). The same is expected for PCBs due to their hydrophobic properties. According to a report from the US Geological Survey, an estimated 162kg of PCB contaminated sediment in the Fox River would be resuspended annually (between lake Winnebago and the DePere Dam), and nearly 143kg would be transported over the DePere Dam (Steuer 1996). It was expected that the quantities going over the DePere Dam would decrease by half every 5 years. It was also reported that approximately 19kg of PCBs are volatized and released into the atmosphere in the same region of the Fox River previously mentioned. Sediment flux and mass sedimentation rates provide insight into mass quantities of transported pollutants over time. A study from 2000 estimated an average HgT mass sedimentation flux of 20ng/cm^2^/year from the Fox River to the Bay for surficial sediment samples collected between 1987 and 1990 (Rossman and Edington 2000). Higher mass sedimentation rates were obtained for PCBs in another study for samples collected in 1984 where the highest rate recorded was 0.189 g/cm^2^/year (Hermanson et al. 1994). The higher sedimentation rates were associated with the sample sites closest to the Fox River, while slower rates were found for samples further out into the Bay.

Solid/liquid distribution coefficients (*K*_d_) are used to indicate the mobility of a contaminant in an environment (CLARC 2017). This provides a simplistic model of sorption for sediment and is crucial to understanding risk of transport. Essentially, *K*_d_ indicates the hydrophobicity of a contaminant where higher values indicate higher hydrophobicity. Thus, a higher distribution coefficient corresponds to higher pollutant concentration in the solid matrices as opposed to the liquid matrices. These values are influenced by a variety of factors such as soil pH, redox conditions, particle size distribution, biological activity, and temperature. Another significant factor influencing PCB mobility in sediments includes organic carbon content, specifically the fractional organic carbon (foc). Distribution coefficients for PCBs directly depend on the organic carbon content, which can be applied as a ratio (*K*_d_/foc) to provide the organic carbon normalized distribution coefficient (Koc)(Baker et al. 1991). While organic carbon data was not collected for this study, it is very likely that PCB samples with higher mobility also have higher organic carbon content. Values above 104 are above the upper limit of *K*_d_, indicating immobility, and values less than 0.1 are below the lower limit, indicating high mobility (Sheppard et al. 2009). The distribution coefficient was calculated for mercury and PCBs (Table 1); however, the calculations applied to PCBs have substantial error associated with the method given that the pore water samples yielded concentrations close to or below the detection limit, further emphasizing the lack of mobility for these compounds. The mercuric ion maintains its high affinity for depositing onto sediment with minimal leaching sediment back into the water. The spatial trend of the HgT sediment concentrations, shown in Fig. 1, reveals high concentration regions along the eastern and central areas of the bay. The samples provide a general depiction to the degree of mercury transport from the Lower Fox River into the bay. The HgT sediment concentrations range from 0.0203 (site ID 14) to 0.460 mg·kg^-1^ (ID 38), with a mean of 0.170 mg·kg^-1^. Detailed statistics for Lower Green Bay Mercury II Concentration Statistics for solid matrix (SM Table 1) and pore water (SM Table 2). Similar trends for PCBs were observed in terms of spatial distribution but did not show significant mobility. The higher PCB concentrations can be found in the sediment near the middle and eastern regions of the bay with minimal amounts found in the pore water. Since the concentrations are comparable to historical results, there is not any evidence that PCBs are being transported in substantial quantities. For PCBs, this behavior can be explained by the low solubility of Aroclor 1242 in water and its affinity for solid materials. Additional sampling along the western side would be needed to have a better understanding of these patterns in the bay.

**Fig. 3:**
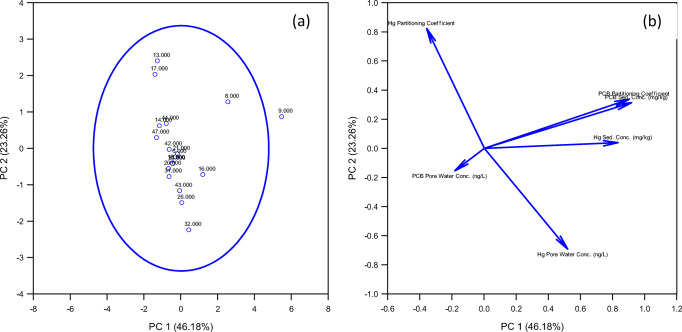
PCA for PCBs and mercury distribution coefficients, pore water, and sediment samples (**a**). The component loading plot shows both the magnitude and direction of the Eigenvalue (**b**)

To further evaluate the data from sediment concentrations, pore water concentrations, and distribution coefficients for both PCBs and mercury, principal component analysis was performed. When plotting the principal components 1 and 2 (Fig. 3), the results show strong correlations between PCB distribution coefficients with PCB sediment concentration to mercury sediment concentrations. For PCBs, the sediment contains the highest concentration in the sample, leading to higher distribution coefficients. While Hg has a high affinity for sediment, the results show that PCBs are much less mobile than Hg. A portion of Hg found in sediment is fixed to the sediment; however, there are other portions that can be exchanged in pore water. In general, both contaminants have a stronger affinity to sediment and do not diffuse in substantial quantities when compared to the sediment concentration; most of the Hg and PCB presence will be contained within the sediment.

## Conclusion

When comparing the concentrations obtained in this study with historic data for mercury, the pore water concentrations found in the bay appear to be higher than what has been found along the lower Fox River. The sediment samples in the bay maintain a considerably lower mercury concentration than historically observed in river sediments. Resuspension is likely the main mechanism of mercury transport which may be due to the sediment being agitated, allowing it to spread from the river to the bay, thus dispersing the mercury contaminated sediment in a larger area. Additionally, this resuspension allows for mercury to leach out of the sediment back into the water where the pollution is then carried by currents. Concentrations of PCBs in sediments from this study show very similar concentrations to historical data and it can be concluded that not much has changed in terms of degradation for PCBs in Green Bay, providing a good example of how stable these pollutants are.

The distribution coefficients from the sediment-pore water lend to a greater understanding of the means in which mercury and PCBs are transported from the Lower Fox River to lower Green Bay. Due to the observation that higher concentrations of these pollutants are found in the sediment than in the water, the assumption that resuspended contaminated sediment is the root cause of mercury and PCB spreading seems credible. These observations agree with the results obtained from PCA, showing trends that favor higher affinity to sediment for both analytes. The lower mercury and PCB concentrations found in the pore water reveal that these contaminants have a higher affinity to solids. However, that does not mean the pollutants will not desorb from the sediment under certain conditions.

## Supplementary Information

ESM 1 (PDF 90.5 kb)

## Data Availability

The datasets used and/or analyzed during the current study are available from the corresponding author on reasonable request.
