# Supplementary material for: Assessment of concentration and distribution of total mercury and polychlorinated biphenyls in Green Bay, Wisconsin, USA
Source: Environ Sci Pollut Res Int. 2021 Sep 29;29(9):13323–32. doi: 10.1007/s11356-021-16417-6 (PMC8803791; doi:10.1007/s11356-021-16417-6)
Supplement: Supplementary file 1 — (PDF 90.5 kb) [file 11356_2021_16417_MOESM1_ESM.pdf]

Assessment of changes in distribution of total mercury and polychlorinated biphenyls in Green Bay, Wisconsin, USA

Marcia R. Silva[1, *], Alice Lecus[1], Chad Haehle [1], David Garman[2] and Shelby Brunner[3]

[1] Water Technology Accelerator (WaTA), University of Wisconsin-Milwaukee, 247 W. Freshwater Way, Milwaukee, Wisconsin, 53204 (USA)

[2] Centre for Infrastructure Engineering, Western Sydney University, 56 Second Avenue, Kingswood, 2747 NSW Australia

[3] UCAR-The University Corporation of Atmospheric Research, Silver Spring, MD, 20910, USA

*Corresponding author: msilva@uwm.edu, phone number: +1-414-326-8285, fax number: +1-414-382-1705.

Supplementary Materials:

Supplementary Material (SM) Table 1: Lower Green Bay Mercury II Concentration Statistics for Solid Matrix

Site ID	Number of Replicates	Mean (mg·kg^{-1})	Stdev (mg·kg^{-1})	Range (mg·kg^{-1})		RSD %[1]
				Min	Max	
6	3	0.164	0.006	0.157	0.168	3.58
8	3	0.244	0.003	0.241	0.246	1.18
9	2	0.456	0.006	0.452	0.460	1.24
13	3	0.131	0.005	0.127	0.136	3.62
14	3	0.0203	0.000	0.0203	0.0204	0.28
16	3	0.428	0.019	0.406	0.439	4.45
17	3	0.0916	0.007	0.0832	0.0964	7.97
18	2	0.149	0.002	0.147	0.150	1.43
20	3	0.168	0.006	0.162	0.173	3.38
21	3	0.186	0.006	0.180	0.192	3.23
26	3	0.108	0.008	0.100	0.115	7.20
27	3	0.0403	0.001	0.0392	0.0414	2.74
31	3	0.106	0.003	0.103	0.109	2.87
32	3	0.109	0.003	0.105	0.111	2.96
33	3	0.120	0.005	0.115	0.124	3.77
38	2	0.460	0.008	0.454	0.466	1.84
42	3	0.117	0.004	0.113	0.120	3.01
43	3	0.115	0.006	0.108	0.119	5.29
44	3	0.112	0.006	0.105	0.115	5.17
47	3	0.0662	0.003	0.0630	0.0699	5.27

[1] RSD% is the relative standard deviation

Supplementary Material (SM) Table 2: Lower Green Bay Mercury II Concentration Statistics for Pore Water

Site ID	Number of Replicates	Mean (ng·L^{-1})	Stdev (ng·L^{-1})	Range (ng·L^{-1})		RSD %[2]
				Min	Max	
6	3	128	12.1	115	139	9.47
8	2	124	15.6	113	135	12.55
9	3	414	32.8	392	452	7.92
13	2	11.9	0.9	11.2	12.5	7.76
14	3	6.39	0.4	5.99	6.87	6.95
16	2	315	111.7	236	394	35.47
17	2	9.48	1.4	8.45	10.5	15.30
18	2	127	24.0	89.1	144	18.93
20	2	115	37.7	88.7	142	32.67
21	3	126	8.6	117	134	6.82
26	2	441	21.2	426	456	4.81
27	3	Below the limit of detection				
31	2	167	39.5	177	200	23.72
32	3	664	9.6	653	671	1.45
33	3	148	8.2	139	155	5.53
38	2	55.7	1.6	54.6	56.8	2.79
42	2	79.8	13.1	56.3	89.0	16.40
43	3	346	26.9	315	365	7.77
44	2	30.5	2.0	29.1	31.9	6.49
47	2	18.5	1.8	17.2	19.8	9.94

[2] RSD% is the relative standard deviation
